# GDE Stability in CO_2_ Electroreduction to Formate: The Role of Ionomer Type and Loading

**DOI:** 10.1021/acscatal.5c02052

**Published:** 2025-05-09

**Authors:** Jose Antonio Abarca, Lucas Warmuth, Alain Rieder, Abhijit Dutta, Soma Vesztergom, Peter Broekmann, Angel Irabien, Guillermo Díaz-Sainz

**Affiliations:** † Departamento de Ingenierías Química y Biomolecular, 685983 Universidad de Cantabria, Avenida de los Castros s/n, Santander 39005, Spain; ‡ 150232 Institute of Catalysis Research and Technology (IKFT), Karlsruhe Institute of Technology (KIT), Hermann-von-Helmholtz-Platz 1, Eggenstein-Leopoldshafen 76344, Germany; § Department of Chemistry, Biochemistry and Pharmaceutical Sciences, 27210 University of Bern, Freiestrasse 3, Bern 3012, Switzerland; ∥ NCCR Catalysis, University of Bern, Freiestrasse 3, Bern 3012, Switzerland; ⊥ MTA−ELTE Momentum Interfacial Electrochemistry Research Group, Eötvös Loránd University, Pázmány Péter sétány 1/A, Budapest 1117, Hungary

**Keywords:** CO_2_ electroreduction, gas diffusion electrode, ionomer, stability, formate

## Abstract

The electrochemical reduction of CO_2_ (ERCO_2_) to formate is a promising decarbonization strategy, yet the long-term stability of gas diffusion electrodes (GDEs) remains a major bottleneck for large-scale implementation and technoeconomic viability. This study systematically investigates the role of catalyst layer (CL) composition in enhancing GDE performance and durability, focusing on ionomer selection, catalyst-to-ionomer ratio optimization, and the use of additives (such as PTFE) to tune the CL hydrophobicity. As a catalyst, (BiO)_2_CO_3_ is used as an active material thanks to its selectivity toward formate. The impact of the ionomer type is evaluated by comparing *Nafion*, a proton-conducting ionomer, with *Sustainion*, an anion-conducting ionomer. While *Nafion*-based GDEs exhibit competitive selectivity toward formate at low ionomer content, with Faradaic efficiencies (FE) around 85%, increasing the ionomer concentration can promote hydrogen evolution reaction (HER), with FEs for H_2_ even exceeding 60%, due to worsened catalyst distribution and the clogging of CO_2_ pathways to the active catalyst sites. In contrast, *Sustainion*-based GDEs effectively suppress HER across all catalyst-to-ionomer ratios, achieving high FEs for formate, in the range of 60–90%. However, even with *Sustainion*, excessive ionomer loading leads to pore clogging, limited CO_2_ accessibility, and decreased formate production. To further enhance stability, PTFE is introduced as an additive alongside *Sustainion*, tuning the hydrophobicity of the CL. By optimizing the amount of PTFE to add, we achieve continuous operation for 24 h, maintaining a high FE for formate (∼85%) and keeping HER below 10%, with formate rates of 8.92 mmol m^–2^ s^–1^ and single-pass conversion efficiencies of 5.81%. Stability studies reveal that *Nafion*- and *Sustainion*-only GDEs suffer from electrolyte flooding over time, which limits the CO_2_ transport and accelerates HER. In contrast, flooding can be prevented on PTFE-modified GDEs, enabling permanent catalyst accessibility and preventing high HER rates. These findings underscore the critical role of CL composition in achieving prolonged GDE stability. By leveraging anion-conducting ionomers and optimizing hydrophobicity, this work provides a pathway toward the scalable deployment of ERCO_2_ in formate technology.

## Graphical abstract

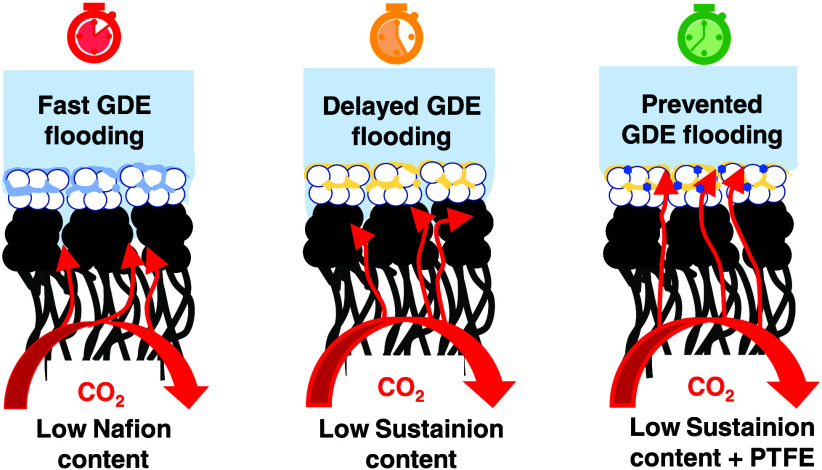

## Introduction

Anthropogenic CO_2_ emissions are a major driver of global warming and climate change. To mitigate these emissions, various strategies have been explored, including the adoption of low-carbon energy sources and improvements in the energy efficiency. Among these approaches, carbon capture and utilization (CCU) has emerged as a promising solution for decarbonizing hard-to-abate industries while enabling the conversion of CO_2_ into value-added chemicals.^1,2^

Electrochemical CO_2_ reduction (ERCO_2_) has gained significant attention as a CCU technology for converting CO_2_ into useful products.^3^ This process involves the electrochemical transformation of CO_2_ into chemicals by applying an external voltage to an electrochemical cell.^4^ In alignment with circular economy principles, ERCO_2_ not only valorizes residual CO_2_ but also enhances the sustainability of industrial processes.^5^ When powered by renewable energy sources, ERCO_2_ enables the storage of intermittent renewable energy in chemical bonds while simultaneously reducing CO_2_ emissions.

A variety of products can be obtained through ERCO_2_, including carbon monoxide (CO), methanol (CH_3_OH), ethanol (CH_3_CH_2_OH), ethylene (C_2_H_4_), methane (CH_4_), and formate/formic acid (HCOO^–^/HCOOH).^6^ This selectivity toward specific products is influenced by several factors, including current density, applied cathode voltage, reaction medium, and electrocatalyst type. Among these products, formate is particularly promising due to its industrial relevance, with recent advancements bringing its large-scale implementation closer to reality.^7^ Research efforts have focused on optimizing catalysts,^8^ reactor designs,^9^ reaction conditions,^10,11^ and electrode fabrication technique,^12^ achieving Faradaic efficiencies (FE) exceeding 90% for formate production.^7^

The core component of the ERCO_2_ technology is the cathode (working electrode), where the CO_2_ reduction reaction occurs.^13^ Among various electrode designs, gas diffusion electrodes (GDEs) have demonstrated superior performance due to their ability to enhance CO_2_ mass transfer.^14^ GDEs feature a porous structure with a catalyst-coated surface, facilitating a well-defined triple-phase boundary where the solid catalyst, liquid electrolyte, and gaseous CO_2_ interact.^15,16^

A typical GDE consists of multiple layers, as presented in Figure 1:

(i) Gas diffusion layer (GDL): the foundation of the GDE, typically composed of hydrophobic, porous, and conductive carbon-based materials.^17^ Some studies have also explored noncarbonaceous alternatives.^18^ The GDL facilitates CO_2_ transport to the catalyst and removes gaseous products from the reaction zone.^19^ It is constructed by depositing a microporous layer (MPL) onto a conductive substrate, usually made of carbon fibers or titanium foam.^20^ The MPL comprises carbon particles bound with a hydrophobic polymer, such as PTFE, ensuring high porosity and hydrophobicity.^17^
(ii) Catalyst layer (CL): the active component of the GDE, deposited onto the GDL via techniques such as sputtering or spray deposition.^14^ In these methods, an ionomer is required to bind the catalyst particles to the MPL, ensuring efficient ion transport across the electrode surface.^21^ Achieving a homogeneous distribution of the CL is crucial for maintaining uniform CO_2_ exposures to active sites, ensuring consistent reactant availability, and stabilizing the local reaction environment.^22^

**Figure 1:**
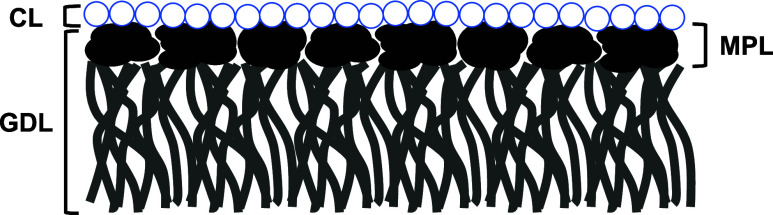
Schematic representation of a typical GDE, highlighting its individual layers and their respective functions.

Despite their high performance, GDEs face significant stability challenges when scaling up the ERCO_2_ technology. The primary degradation mechanisms include:

(i) Catalyst deactivation: detachment, dissolution, or alteration of the catalyst under reaction conditions.^23^
(ii) Precipitation of carbonate and bicarbonate salts: formation of insoluble salts under alkaline conditions, leading to electrode clogging and restricted CO_2_ access.^24^
(iii) GDE flooding: changes in wettability promote electrolyte infiltration, blocking pores, and increasing the competitive hydrogen evolution reaction (HER).^25^

Addressing these degradation mechanisms is critical for improving the durability and scalability of GDE-based ERCO_2_ systems and their technoeconomic evaluation.^26^

The rational design of catalytic materials plays a pivotal role in improving both activity and stability of ERCO_2_.^27^ Bismuth-based catalysts are particularly noteworthy among various catalysts due to their high selectivity toward formate. Bi_2_O_3_ has been widely studied^28^ but its reduction and conversion to other Bi oxidation states under reducing conditions lead to increased hydrogen evolution over time.^8^ More specifically, Bi_2_O_3_ converts into (BiO)_2_CO_3_ upon contact with CO_2_ in a moist state. Thus, to avoid this process, (BiO)_2_CO_3_ itself is used as the catalyst in this case.^29^

In zero-gap configurations, the precipitation of (bi)­carbonate salts is particularly problematic, as the absence of a liquid catholyte promotes salt precipitation.^24^ Strategies to address this issue include modifying anolyte composition by the introduction of alternative cations, such as Cs^+^, to form more soluble salts compared to conventional K^+^ ^30^ and employing acidic anolytes, such as K_2_SO_4_ at pH 1, to prevent salt deposition on the GDE surface.^31^

Even when salt deposition is mitigated, GDE flooding remains a significant challenge, particularly for long-term stability.^20^ Over time, changes in the hydrophobicity and wettability of the GDE allow the catholyte to infiltrate deeper into the electrode, clogging the porous structure of the GDL.^32^ This infiltration impedes CO_2_ diffusion to the CL, hindering the electrode’s performance. Several studies^15,25,33–38^ have highlighted this issue and proposed various solutions. One strategy involves optimizing operational conditions, such as maintaining a controlled pressure difference between the CO_2_ gas inlet and the catholyte side, with the GDE acting as a barrier. This pressure control can help limit catholyte penetration into the GDE structure.^38^ Other approaches focus on tailoring the GDE composition to optimize its wettability and hydrophobicity, thus enhancing operational stability. For instance, some researchers recommend maintaining the hydrophobicity of the CL to preserve the triple-phase boundary and prevent liquid penetration.^34,36^ An alternative approach is the adjustment of the GDE composition to facilitate the drainage of infiltrated liquid, thereby mitigating flooding and maintaining performance.^39^ Consequently, optimizing the CL composition is critical for effectively managing GDE flooding and ensuring stable long-term operation.

The ionomer plays a crucial role in the composition of the CL, as it is the material that binds the catalyst to the MPL, facilitating ionic conduction in this layer.^40^ Traditional proton-conductive ionomers, such as *Nafion*, with a high transference number for H^+^ ions, are widely used in ERCO_2_ for formate production.^21^ However, recent studies have explored anion-conductive ionomers, such as *Sustainion* and Fumion, as alternatives.^41^ These ionomers differ in their ion conduction mechanism and their influence on GDE wettability.^42^ Additionally, the catalyst-to-ionomer ratio significantly affects the GDE performance, requiring optimization to balance active site exposure, adhesion stability, and ionic conductivity.^43^ Since GDE flooding remains a major barrier to long-term operation, optimizing CL composition is critical for extending the electrode lifespan and achieving industrial stability. Beyond ionomers, other polymeric additives, such as poly­(tetrafluoroethylene) (PTFE), can be incorporated into the CL formulation to adjust surface hydrophobicity, influencing the overall stability and performance of the GDE.

This study investigates the influence of ionomer type (*Nafion* vs *Sustainion*), catalyst-to-ionomer ratio, and additional hydrophobic polymers like PTFE on GDE stability in ERCO_2_ to formate, employing a (BiO)_2_CO_3_ active catalyst phase. A laboratory-scale CO_2_ electrolyzer is employed for continuous operation, and the physicochemical properties of fresh and used GDEs are analyzed to assess stability.

## Results and Discussion

### Effect of the Catalyst–Ionomer Ratio on the ERCO_2_ Performance

This section presents an experimental analysis of the CL composition in GDEs, focusing on the mass ratio between the catalyst (the synthesized (BiO)_2_CO_3_) and ionomer. A fresh GDE is subjected to XRD analysis to determine the crystal structure of the catalyst. In this sense, the as-prepared GDEs exhibit characteristic reflections corresponding to orthorhombic (BiO)_2_CO_3_ (Figure S1a), along with background signals associated with the GDE. A broadening of the diffraction peaks is observed, presumably due to the small crystallite size, which is further supported by STEM images (Figure S1b) showing a flakelike, anisotropic morphology. Two ionomers are investigated: *Nafion* (a proton-conducting ionomer) and *Sustainion* (an anion-conducting ionomer). Their effect on the ERCO_2_ to formate is evaluated across various catalyst–ionomer ratios, ranging from 90 to 10 to 30–70 (wt %) while maintaining a constant catalyst loading of 0.75 mg cm^–2^ to make a rigorous comparison. This catalyst loading has been widely used in previous works, serving as a reference for this work.^10,12,44^ Each experiment is conducted for 90 min, focusing on the FE as the primary figure of merit. Initially, the (BiO)_2_CO_3_–*Nafion* ratios are evaluated, as *Nafion* has been widely employed in previous studies.^10,12^ These results are shown in Figure 2.

**Figure 2:**
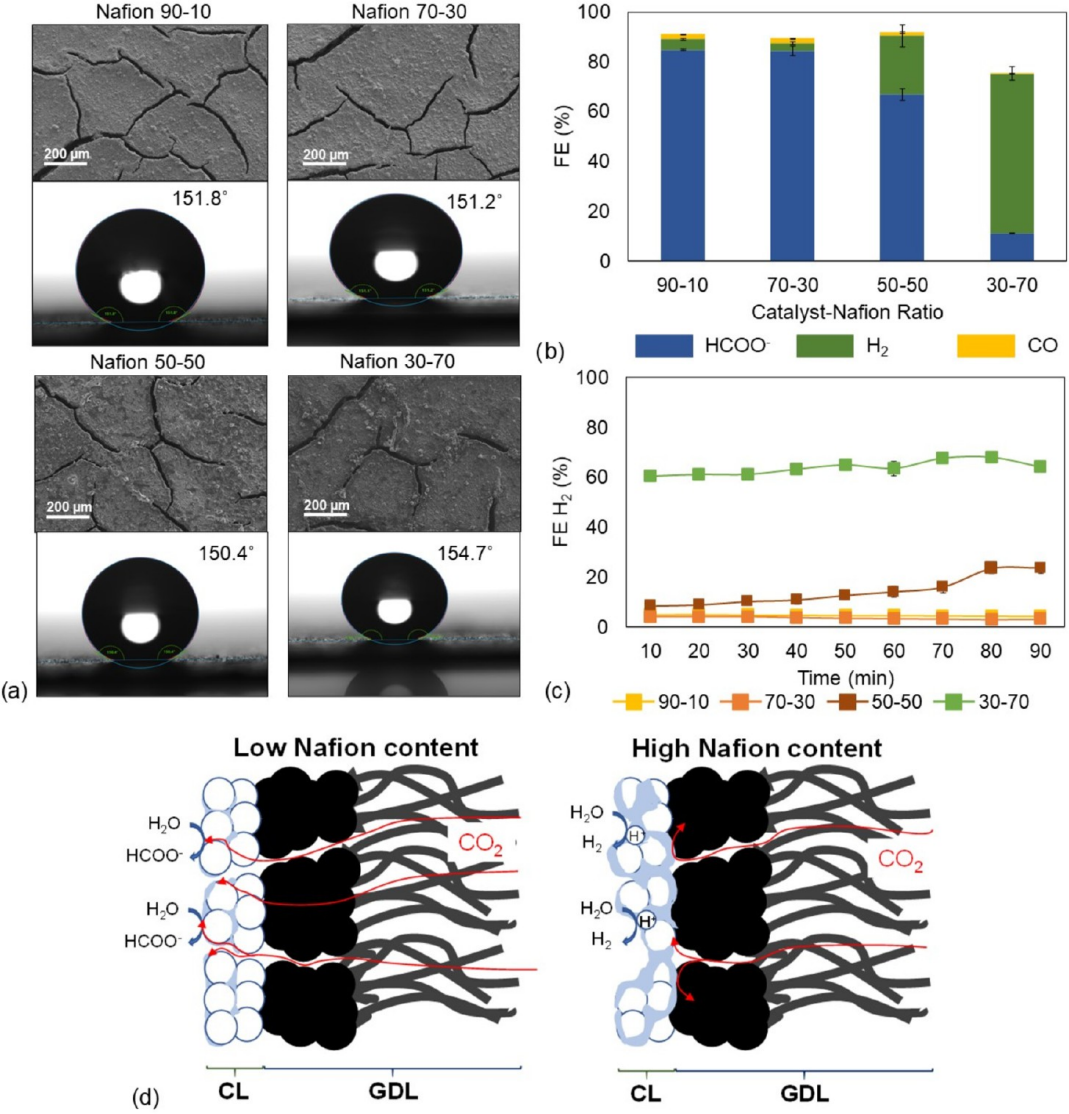
(a) Top-down SEM images and water contact angle measurements for the fabricated GDEs with Nafion as the ionomer; (b) FE for formate, hydrogen, and carbon monoxide at different catalyst–Nafion ratios; (c) FE H_2_ monitoring over the experimental time; and (d) scheme of the different effects of the Nafion loading on the GDE functioning.

Figure 2a shows the structural characterization of the GDEs fabricated with *Nafion* as the ionomer. First, it can be observed that increasing the catalyst–*Nafion* ratio has little significant effect on the surface hydrophobicity, as the water contact angles remain high in all cases. On the other hand, the surface homogeneity is notably affected by the increasing ionomer content, transitioning from a more-or-less evenly distributed catalyst layer (90–10 (BiO)_2_CO_3_–*Nafion* ratio) to an inhomogeneous surface where significant aggregation of the catalyst particles can be observed, e.g., in the case of the 30–70 or 50–50 catalyst–ionomer ratio.

Evaluating the ERCO_2_ to formate performance, Figure 2b reveals a clear trend: as the ionomer mass loading increases, there is a substantial reduction in formate FE, decreasing from 84.8% at the 90–10 ratio to just 11.1% at the 30–70 ratio.^45^ In parallel, the H_2_ FE increases, as shown in Figure 2c. Notably, at lower *Nafion* content (90–10 and 70–30 ratios), the H_2_ FE remains consistently low, around 3% throughout the 90 min experiment. However, for the 50–50 ratio, a significant increase in H_2_ FE to approximately 24% is observed at about 70 min, which may indicate failure due to flooding. Conversely, the 30–70 (BiO)_2_CO_3_–*Nafion* GDE shows consistently high H_2_ FE values, exceeding 60% from the beginning of the experiment. Taking into account the high FEs toward H_2_, it should be noted that these could be underestimated, as the H_2_ concentration detected by the GC may exceed the calibration limit, as well as some H_2_ losses in the direction of the solution phase. This behavior suggests that higher *Nafion* loadings promote the HER while inhibiting ERCO_2_ to formate. In terms of the formate production rate (Table S1), a clear decrease is observed with increasing *Nafion* content: from 8.8 mmol m^–2^ s^–1^ for compositions with low *Nafion* content (ratios 90–10 and 70–30) to only 1.16 mmol m^–2^ s^–1^ for the highest *Nafion* ratio. A similar trend is observed for the SPCE performance, where the conversion efficiency decreases from 5.91% at low *Nafion* content to 0.78% for the 30–70 (BiO)_2_CO_3_–*Nafion* composition.

This effect can be attributed to different factors related to the ionomer. First, the excessive presence of the ionomer, in this case, *Nafion*, hinders the mass transfer of CO_2_ to the active sites of the catalyst, as the possible CO_2_ pathways can be clogged,^43^ as shown in the scheme of Figure 2d. Moreover, the poor lateral catalyst distribution reduces the available catalyst surface area to form the three-phase boundary as well as limiting the transport of reaction intermediates (e.g., CO_2_, CO_2_^–^, or HCO_3_^–^) toward the catalyst’s active sites. Additionally, higher *Nafion* content enhances H^+^ transport due to the proton-conductive nature of this ionomer. All of these factors favor the HER against the ERCO_2_ to formate in those GDEs in which the *Nafion* content surpasses the 50% ratio to the catalyst.

The same evaluation is performed using an anion-conductive ionomer, *Sustainion*, as shown in Figure 3.

**Figure 3:**
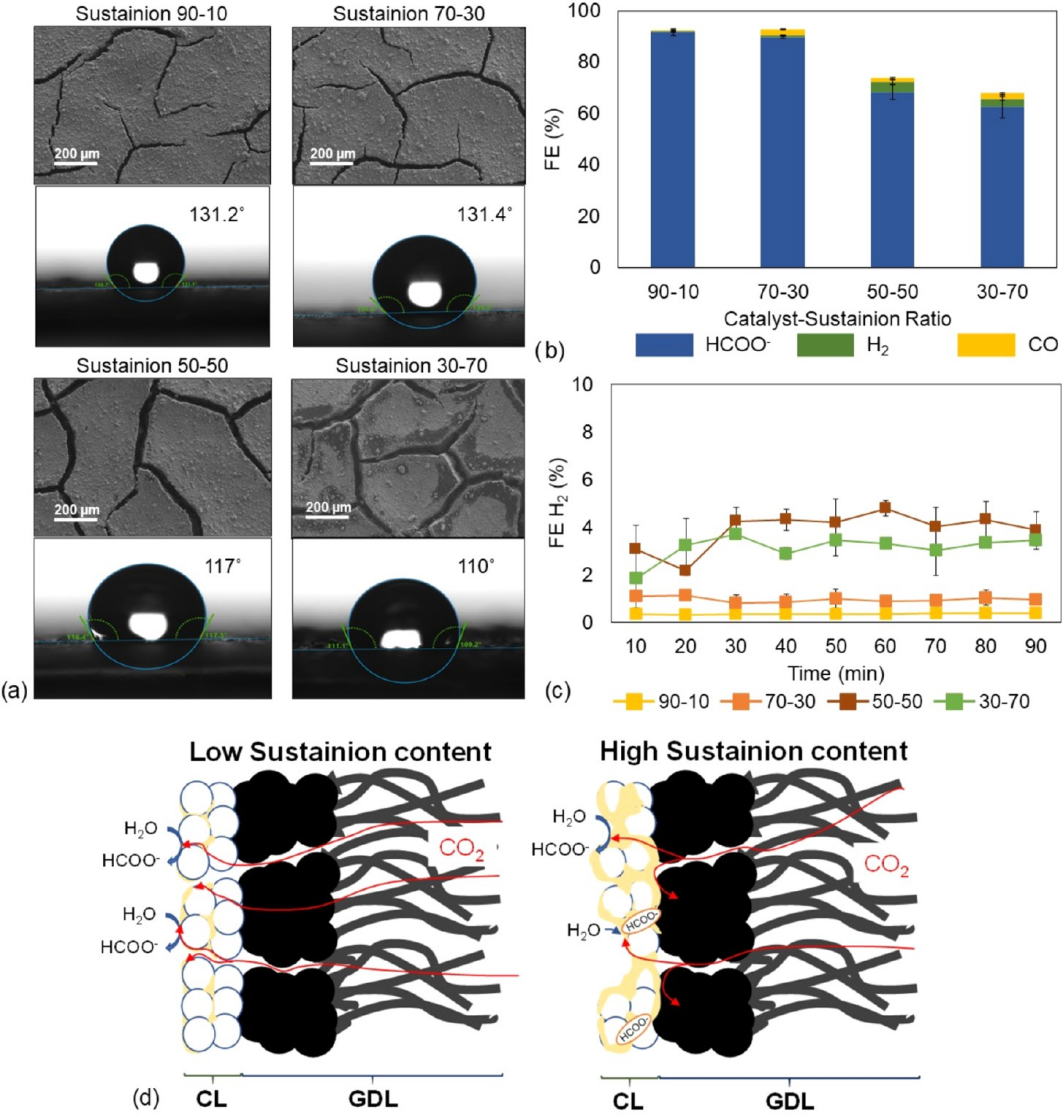
(a) Top-down SEM images and water contact angle measurements for the fabricated GDEs with Sustainion as the ionomer; (b) FE for formate, hydrogen, and carbon monoxide at different catalyst–Sustainion ratios; (c) FE H_2_ monitoring over the experimental time; and (d) scheme of the different effects of the Sustainion loading on the GDE functioning.

The SEM top-down images reveal an effect similar to that observed in *Nafion*-based GDEs: as the ionomer loading increases, the lateral distribution of the catalyst deteriorates (Figure 3a). Additionally, an increase in the *Sustainion* ratio leads to larger crack sizes, with widths increasing from 15–25 μm in the 90–10 ratio GDE to 40–55 μm in the (BiO)_2_CO_3_–*Sustainion* 30–70 ratio. Furthermore, the change in the ionomer content also affects hydrophobicity; higher *Sustainion* amounts in the CL result in lower water contact angle values, indicating a less hydrophobic GDE surface.

In the case of the *Sustainion*-based GDE ERCO_2_ performance, the most notable effect is the suppression of hydrogen generation across all CL compositions, with H_2_ FE remaining between 0.5 and 5%, as shown in Figure 3b,c. The anion-conducting nature of *Sustainion* effectively prevents H^+^ transport within the CL, thereby inhibiting the HER and promoting formate production.^41^ As seen in Figure 3a, high formate FEs exceeding 90% are achieved for the 90–10 and 70–30 catalyst–*Sustainion* ratios. However, as the *Sustainion* content increases, there is a significant decrease in formate FE, with values dropping to 68 and 62% for the 50–50 and 30–70 ratios, respectively. On the other hand, when *Sustainion* is used as the ionomer component, the formate production rates remain high across all compositions, as shown in Table S1. In particular, the GDE with a (BiO)_2_CO_3_–*Sustainion* ratio of 90–10 achieves a production rate of 9.46 mmol m^–2^ s^–1^. SPCE results follow a similar pattern with conversion efficiencies consistently above 4%, with a peak of 6.38% for the same 90–10 ratio.

This excessive amount of anion-conductive ionomer negatively impacts performance, as it can clog the porous structure of GDE limiting the CO_2_ access to the catalyst and retain a large quantity of reaction intermediates or even formate anions within its structure,^46^ as presented in Figure 3d. With more ionomer active sites available for interaction, the desorption rate of reduction products is reduced, ultimately limiting the overall ERCO_2_-to-formate conversion efficiency.

In both cases, regardless of the type of ionomer, a higher ionomer loading impairs ERCO_2_-to-formate conversion. However, the underlying mechanisms for the performance loss differ, owing to their distinct abilities to conduct different ionic species. Notably, the best results for both ionomers are observed at a (BiO)_2_CO_3_–ionomer ratio of 90–10, with *Sustainion* showing a slightly superior performance. Using *Sustainion* as the ionomer achieves a formate FE of 91.6% and a 9.5 mmol m^–2^ s^–1^ production rate while effectively suppressing the HER.

### Impact of PTFE as an Additive to *Sustainion* on the ERCO_2_ Performance

The analysis of different CL compositions has shown that using *Sustainion* as the ionomer enhances ERCO_2_ by almost completely suppressing the HER. In this context, the 90–10 catalyst–ionomer ratio achieves the highest formate FE at 91.6%. Building on this, the next step is to investigate the effect of incorporating PTFE as an additive in the CL to assess how modifications in CL hydrophobicity influence formate conversion performance. Therefore, different *Sustainion*–*PTFE* proportions are studied while maintaining the catalyst mass ratio at 90–10 with respect to the rest of the components of the catalytic ink (binder+additive), each GDE named catalyst–*Sustainion–PTFE* ratio, with the electrolysis results presented in Figure 4.

**Figure 4:**
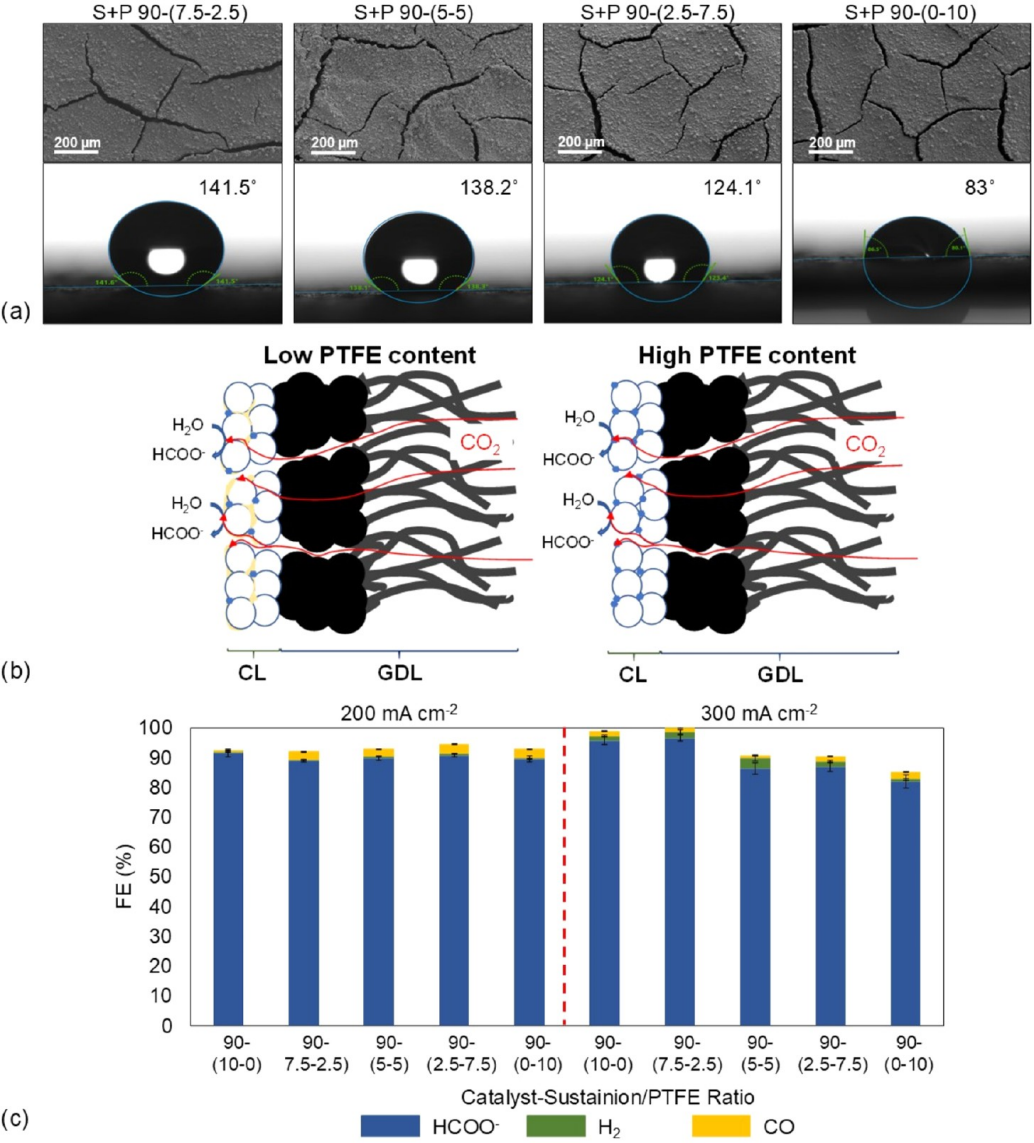
(a) Top-down SEM images and water contact angle measurements for the fabricated GDEs with *Sustainion–PTFE* as the binder; (b) scheme of the different effects of the PTFE loading on the GDE functioning; and (c) FE results for different *Sustainion–PTFE* ratios, maintaining an overall catalyst–ionomer of 90–10 ratio at −200 and 300 mA cm^–2^.

The top-down SEM images (Figure 4a) reveal that CL homogeneity and catalyst distribution remain consistent across all samples, exhibiting similar cracked structures. This result is expected, as the overall catalyst–ionomer ratio is maintained at a constant across all fabricated GDEs. However, the hydrophobicity of CL is significantly influenced by the addition of PTFE, allowing for tailored wetting properties.

For the 7.5–2.5 *Sustainion–PTFE* proportion, the hydrophobicity increases relative to the pure *Sustainion*-based GDE (Figure 3), as indicated by an increase in the water contact angle from 131 to 141̊. A similar hydrophobicity enhancement is observed for the 50/50 ratio. However, when PTFE is used without the presence of any extra binder, the water contact angle decreases to 83̊, indicating a more hydrophilic behavior. The reason behind this decrease in hydrophobicity can be understood by analyzing the scheme in Figure 4b. It shows that when PTFE is used without a binder, it is deposited in the form of particles, which exposes a high area of the catalyst, reducing the surface hydrophobicity and facilitating water penetration into the CL. In contrast, when *Sustainion* is used together with PTFE, its even distribution, due to its polymeric form, increases the surface hydrophobicity, preventing electrolyte penetration into the CL.

Regarding the effect of adding PTFE on the ERCO_2_ to formate (Figure 4c), GDEs varying *Sustainion–PTFE* proportions are tested for 90 min at a current density of −200 mA cm^–2^. In all cases, the formate FE remains around 90%, with negligible H_2_ production, indicating no significant differences between the compositions. However, the presence of PTFE results in a slight increase in CO production, with CO FEs around 3%, compared to just 0.5% when PTFE is absent. In addition, the production rates obtained, ranging from 9.3 to 9.5 mmol m^–2^ s^–1^ (Table S1), position these GDEs within the range of previously reported values (8.33–10.01 mmol m^–2^ s^–1^), confirming their strong performance in the electroreduction of CO_2_ to formate.^10,12,44^ Similarly, the SPCE achieves conversion efficiencies of approximately 9.4%, which also fall within the previously reported range of 5.6–6.7%.

Since no significant changes in ERCO_2_ performance are detected at −200 mA cm^–2^, the GDEs are tested under more demanding conditions by increasing the current density of up to −300 mA cm^–2^. Under these conditions, increasing the PTFE content leads to a decrease in formate FE, accompanied by a slight increase in H_2_ production as the GDE hydrophobicity is reduced.

The highest formate production is achieved with a *Sustainion–PTFE* ratio of 7.5–2.5, reaching a maximum FE of 96.5%. This improvement may be attributed to the optimization of CL hydrophobicity, which facilitates ERCO_2_-to-formate conversion under these conditions.^47^

This increased hydrophobicity recorded for the (BiO)_2_CO_3_–*Sustainion–PTFE* 90-(7.5–2.5) GDE, compared to the *Sustainion* 90–10, positively impacts ERCO_2_ to formate, as it facilitates the repulsion of liquid electrolytes while trapping gas within the CL, facilitating the CO_2_ mass transport.^48^ Therefore, by adjusting the hydrophobicity, it is possible to control the volume of gas and liquid within the CL, and achieving an optimal balance between the two can significantly improve the ERCO_2_ reaction.

### Effect of CL Composition on the GDE Stability

As demonstrated in the previous sections, the composition of the CL, including the ionomer type, catalyst–ionomer ratio, and additive inclusion, significantly affects the ERCO_2_-to-formate conversion. The next step is to evaluate how different CL compositions impact the stability of the GDEs over extended operation. To this end, three high-performing compositions from previous studies are selected: (i) (BiO)_2_CO_3_–*Nafion* 70–30 ratio, which also serves as the reference for previous studies, (ii) (BiO)_2_CO_3_–*Sustainion* 90–10 ratio, and (ii) (BiO)_2_CO_3_–*Sustainion–PTFE* 90-(7.5–2.5) ratio. These GDEs are tested under identical conditions for 8 h, and the results are presented in Figure 5.

**Figure 5:**
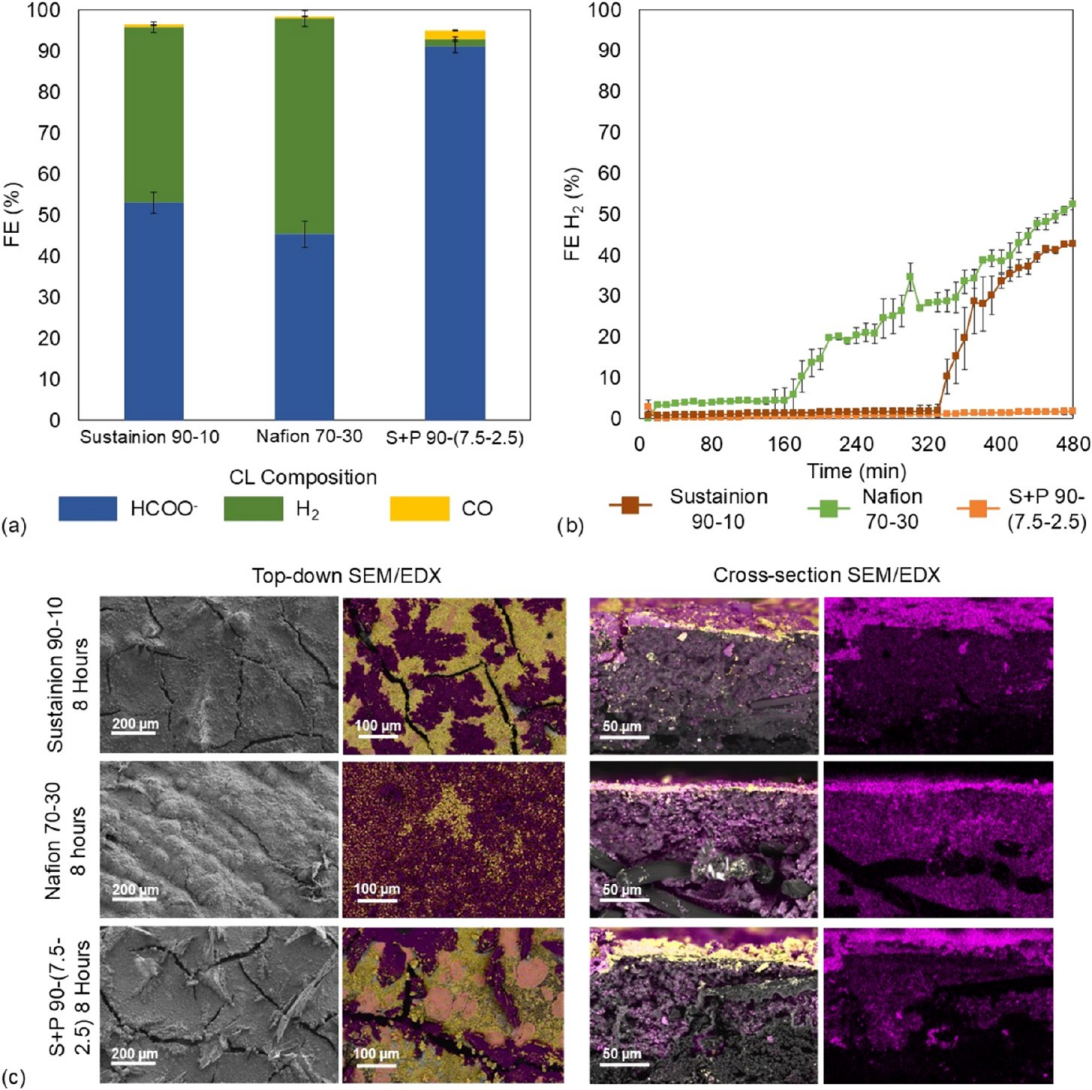
(a) FE results for different CL compositions, (b) FE H_2_ monitoring over 8 h of electrolysis, and (c) top-down and cross-sectional SEM/EDX images of the GDEs after 8 h of electrolysis; yellow = Bi; pink = K.

Among the tested compositions, only the *Sustainion–PTFE*-based GDE 7.5–2.5 ratio maintains a high formate FE, retaining 91.1% after 8 h of operation (Figure 5a), with a formate production rate of 9.44 mmol m^–2^ s^–1^ and an SPCE of 6.35% (Table S1). In contrast, the other two compositions show a significant decline in formate FE compared to their 90 min performance. For (BiO)_2_CO_3_–*Sustainion* 90–10 ratio, the FE drops from 91.6 to 53.1% and the formate rate is reduced from 9.5 to 5.52 mmol m^–2^ s^–1^, while for (BiO)_2_CO_3_–*Nafion* 70–30 GDE, the FE decreases from 84.3 to 45.4% and the production formate rate decreases from 8.74 to 4.70 mmol m^–2^ s^–1^. In the case of the GDE catalyst–*Nafion* 70–30, a sudden increase in FE toward H_2_ is observed after approximately 160 min, indicating the onset of erratic behavior. Meanwhile, for the GDE catalyst–*Sustainion* 90–10, its failure or the beginning of improper behavior is delayed until around 330 min.

This performance decline suggests GDE degradation, leading to a loss of ERCO_2_ activity over time. Regarding the formation of byproducts, H_2_ emerges as the primary competing reaction during electrolysis. Since GC measurements are taken every 10 min, the evolution of H_2_ FE is continuously monitored. Figure 5b illustrates the time-dependent variation of the H_2_ FE, revealing notable trends. For (BiO)_2_CO_3_–*Nafion* 70–30, there is a sudden increase in H_2_ FE around 160 min, reaching a final value of 52.5%, while a similar increase is observed for (BiO)_2_CO_3_–*Sustainion* 90–10 at approximately 330 min up to 42.7%. In contrast, the H_2_ FE for the (BiO)_2_CO_3_–*Sustainion–PTFE* 90-(7.5–2.5) GDE remains stable throughout the entire experiment, with values lower than 1.8%.

The observed increase in H_2_ production, along with the overall reduction in formate yield, can be attributed to GDE failure due to electrode flooding. This flooding effect is caused by changes in hydrophobicity and wettability over time as charge accumulates. Flooding occurs abruptly, as indicated by the H_2_ FE profiles. When the pores become flooded, the transport of CO_2_ to the reaction zone is hindered, favoring the HER over formate production.

GDE flooding can be assessed by using various characterization techniques, with one of the most common methods being the cross-sectional EDX analysis of K^+^ (Figure 5c). This technique provides insights into electrolyte penetration depth within the GDE structure. Additionally, top-down SEM analysis reveals surface modification that occurs during electrolysis. These images reveal significant surface alterations following electrolysis. Notably, in the case of the (BiO)_2_CO_3_–*Nafion* 70–30 GDE, severe potassium salt accumulation, nearly completely covering the electrode surface —even burying the cracks—is visible after electrolysis. This is attributed to the cation-conducting nature of *Nafion*, which facilitates K^+^ accumulation and the subsequent formation of potassium carbonate and bicarbonate.^21^ In the other cases, the observed precipitate formation is less extensive. In the (BiO)_2_CO_3_–*Sustainion* 90–10 GDE, a higher presence of K^+^ is observed on the surface, partially covering the cracks. However, for the (BiO)_2_CO_3_–*Sustainion–PTFE* 90-(7.5–2.5) GDE, salt deposition appears more localized, concentrating around the cracks without fully covering them. This may be linked to the greater hydrophobicity maintained throughout CO_2_ electrolysis, and also the presence of cracks on the GDE surface may cause an adhesion effect of the electrolyte inside the cracks and the underlying fibrous structure.

In the cases of *Nafion* 70–30 and *Sustainion* 90–10 cross-sectional images, higher K^+^ concentrations are observed throughout the electrode structure, covering almost the entire cross-sectional surface of the GDE. In contrast, for (BiO)_2_CO_3_–*Sustainion–PTFE* 90-(7.5–2.5), the highest concentration of K is observed mainly in the CL, with lower intensity in the cross-sectional area. Potassium is not detected throughout the entire cross section of the GDE, suggesting that pore flooding has been prevented or at least delayed during the 8 h electrolysis.

Furthermore, it is also worth noting that the catalyst itself undergoes certain changes during stability experiments lasting several hours. The SEM images (Figure 6a) in all three cases reveal a reconstruction of the (BiO)_2_CO_3_ catalyst, which initially has a nanosheet morphology. Meanwhile, in the GDEs used, changes in the morphology of the catalyst can be observed, leading to the formation of nanoflowers. The rearrangement of the catalyst facets in these nanoflowers exposes more active sites to the electrolyte, allowing them to carry out the ERCO_2_ to formate.^49^ Despite its reconstruction into a nanoflower-like shape, the size of the nanoflowers differs depending on the composition of the CL. While the GDEs with *Nafion* 70–30 and *Sustainion* 90–10 compositions exhibit well-formed and larger nanoflowers, with diameters ranging from 1.3 to 1.7 μm, the GDE with (BiO)_2_CO_3_–*Sustainion–PTFE* 90-(7.5–2.5) shows smaller and less defined structures, the size of which varies between 0.55 and 0.85 μm. This may suggest that the restructuring process has not been fully completed in this case, unlike in the other two. On the other hand, the composition remains constant, meaning this reconstruction does not imply a change in the oxidation state, with (BiO)_2_CO_3_ remaining the predominant active material, confirmed by Raman spectroscopic analysis performed before and after electrolysis for the three GDEs, as presented in Figure 6b–d.

**Figure 6:**
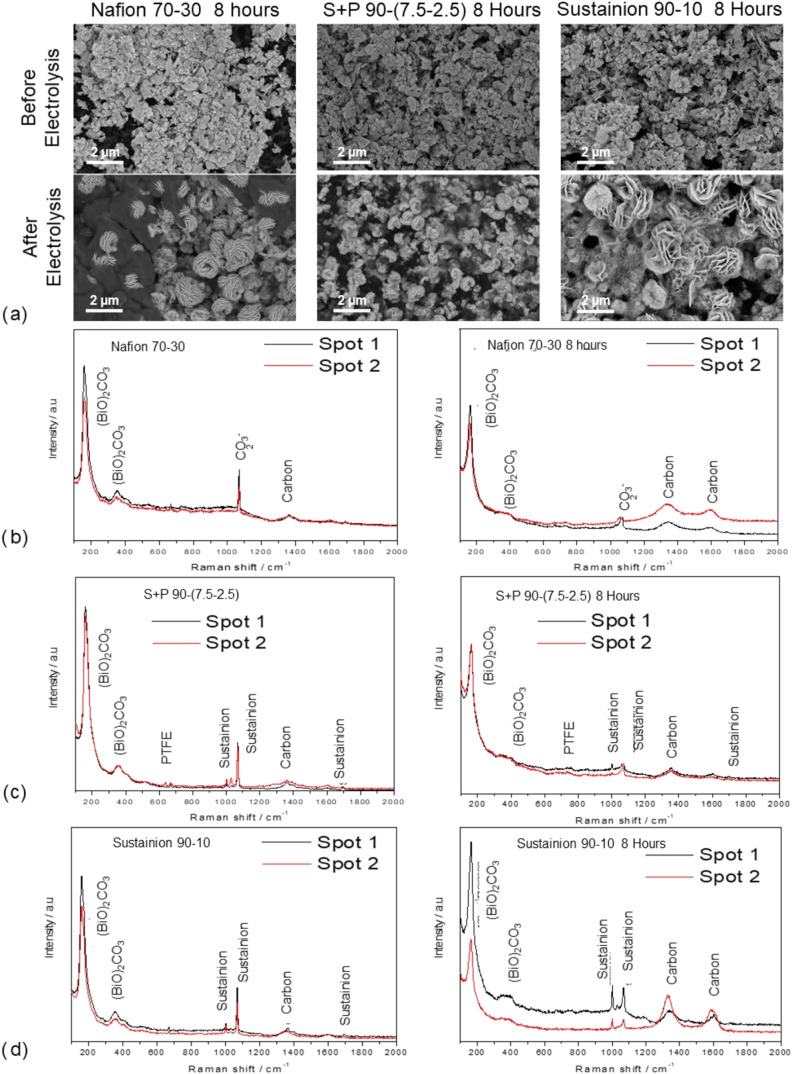
(a) SEM images at a 10K magnification to evaluate the catalyst structure before and after 8 h of electrolysis, and Raman spectra before and after electrolysis for (b) catalyst–*Nafion* 70–30, (c) catalyst–*Sustainion*–*PTFE* 90-(7.5–2.5), and (d) catalyst–*Sustainion* 90–10, with (BiO)_2_CO_3_ as the catalyst.

In addition, electrochemical impedance spectroscopy (EIS) measurements were performed at – 0.8 V vs Ag/AgCl using the same experimental setup, within a frequency range from 10 kHz to 0.1 Hz. The Nyquist plots (Figure S2) show clear differences between the GDEs. The *Nafion*-based GDE displays the largest semicircle, corresponding to the highest charge-transfer resistance and limited mass transport, likely due to higher ionomer loading and ineffective CO_2_ diffusion.^50^ In contrast, the *Sustainion* 90–10 GDE shows a smaller semicircle and a low-frequency tail, indicating lower transfer resistance and a mass-transport-limited regime.^51^

The best performance is seen in the *Sustainion*–*PTFE* 90–(7.5–2.5) GDE, which presents the smallest semicircle and a similar mass transport tail. Its Nyquist curve lies below the others, suggesting improved CO_2_ transport, likely due to better electrolyte management afforded by PTFE addition.^52^

Other factors, such as the type of catalyst or substrate, can affect the stability of the GDE. In this regard, GDEs with the same CL composition have been tested, replacing the (BiO)_2_CO_3_ catalyst with Bi_2_O_3_. After an 8 h test, the FE toward formate remains at high values, reaching up to 84%, while the FE toward H_2_ is kept below 15% at all times (Figures S3 and S4). Additionally, different substrates are used, specifically AvCarb 50% PTFE-treated and carbon cloth, both of which result in FE values toward formate similar to those obtained for the GDE supported on Sigracet 36 BB. In the case of AvCarb, the FE toward formate reaches 87.5%, with an FE toward H_2_ of 7.5%. For the carbon cloth, these values are improved, achieving an FE toward formate of 89.2% and an FE toward H_2_ of only 0.35% (Figures S5 and S6). Therefore, it is also demonstrated that a stable CL composition, such as catalyst–*Sustainion–PTFE* 90-(7.5–2.5), exhibits similar stability despite changes in the type of catalyst or substrate.

### GDE Stability from Hours to Days

The stability of the GDE is compromised by a series of deactivation mechanisms. As observed, electrode flooding is one of the most significant factors during long operation times. Based on this, an analysis of the optimal conditions determined in previous sections is proposed, using (BiO)_2_CO_3_ as the catalyst, with a Catalyst–*Sustainion–PTFE* 90-(7.5–2.5) ratio and Sigracet 36 BB as the substrate, to demonstrate the possibility of extending the operational time scale. Specifically, the goal is to improve durability from approximately 3 h (observed in the initial case, where the CL composition was based on *Nafion* 70–30, following previous studies) to at least 1 day at a constant −200 mA cm^–2^ current density.

To achieve this, 24 h experiments are conducted, continuously monitoring various variables such as FE toward H_2_ to assess GDE flooding, cathode voltage, working electrode resistance, catholyte, CO_2_ inlet pressure, and GDE perspiration in the CO_2_ outlet stream. Additionally, the final catholyte sample is analyzed to quantify the formate produced.

A high FE toward formate is maintained throughout the 24 h experiment, reaching 84.7% and effectively maintaining a production rate of 8.92 mmol m^–2^ s^–1^ and an SPCE of 5.81%. This indicates that the GDE’s performance remains practically unchanged, with only a 7% decrease in the FE and a 6% reduction in the production rate to formate and SPCE compared to the results obtained in the CL composition screening.

On the other hand, Figure 7 presents two closely related measured variables: the FE toward H_2_ and the conductivity of the perspiration, which refers to the condensation of a liquid drop in the back of the GDE, indicating possible electrolyte permeation through the GDE. As observed, the FE toward H_2_ remains below 10% throughout the experiment. However, a noticeable increase coincides with an increase in the conductivity measured in the perspiration. This increase is minor and stabilizes at constant values for the remainder of the experiment. This suggests that a liquid droplet may have condensed on the back of the GDE and been carried away by the CO_2_ stream to the conductivity trap, leading to the observed increase in FE toward H_2_. Since no additional droplets formed during the rest of the experiment, both conductivity and FE toward H_2_ remained within a stable range. Moreover, the FE toward CO is also monitored, with a trend inverse to the FE of H_2_, showing higher values (up to 1.5%) at the beginning and decreasing almost to zero after hour 7. Additionally, other key variables, such as the pressure difference between the CO_2_ inlet and the catholyte, the resistance of the working electrode, and the cathode potential, were continuously monitored throughout the 24 h experiment. These parameters remained stable without abrupt changes that could indicate potential GDE degradation, as shown in Figure S7. This supports the long-term stability of (BiO)_2_CO_3_–*Sustainion*–*PTFE* 90-(7.5–2.5) GDE under the tested conditions.

**Figure 7:**
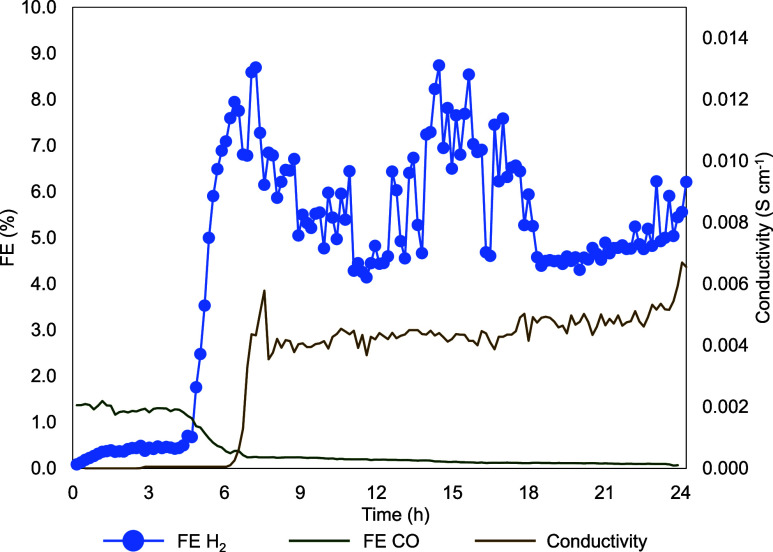
H_2_ and CO FE and perspiration conductivity evolution over the 24 h test.

Furthermore, the GDE used during the 24 h electrolysis is characterized to identify possible physicochemical changes in the electrode that could affect its stability over longer periods of operation.

As seen in Figure 8a, the surface of the GDE does not exhibit significant morphological alterations, maintaining its structure with cracks that aid in electrolyte management. The EDX image reveals the deposition of K^+^ salts on the surface with higher intensity around these fractures in the material. However, due to the dissolution of a large part of these salts in the liquid electrolyte, a large number of active catalyst sites remain accessible.

**Figure 8:**
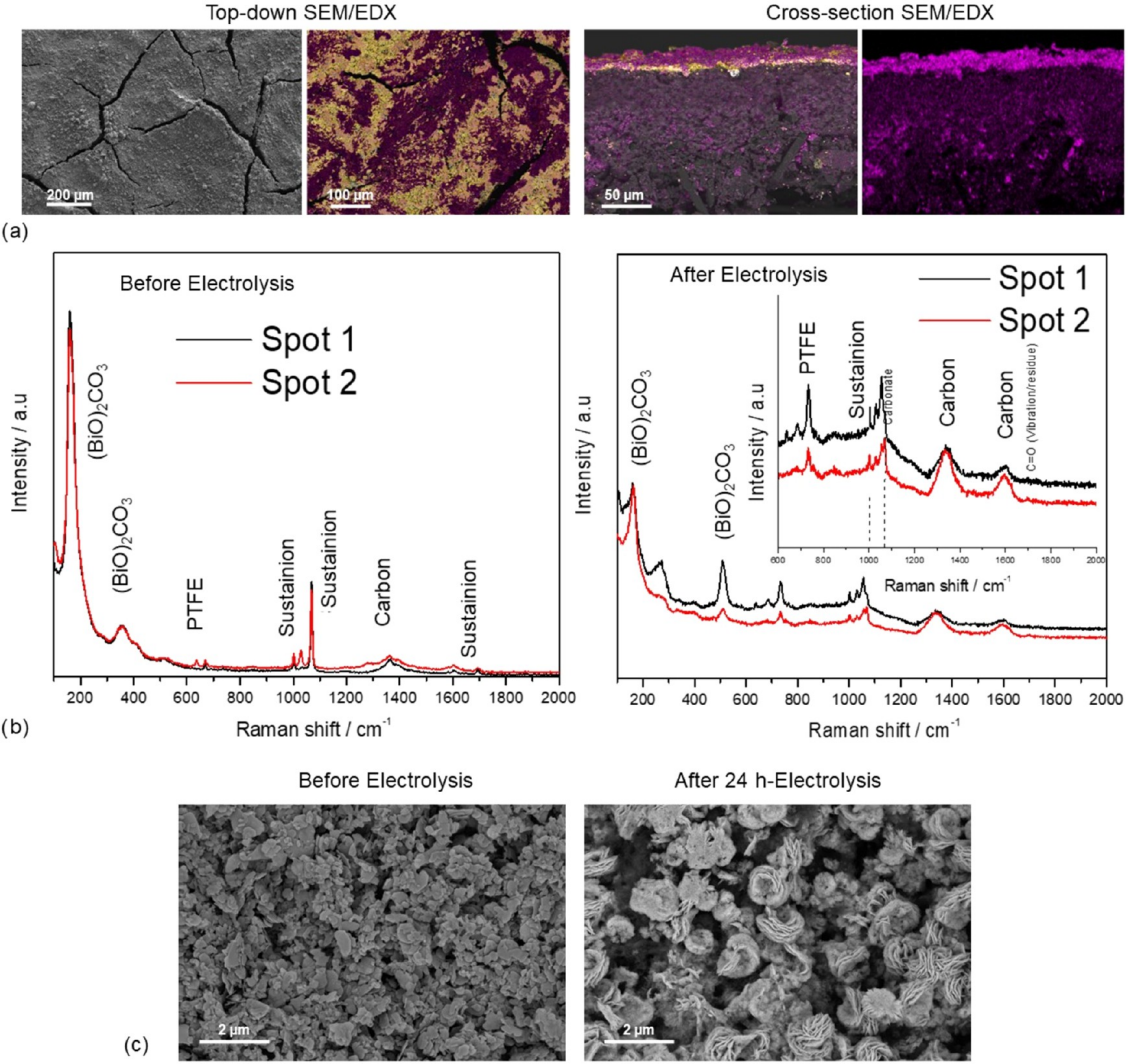
(a) Top-down and cross-sectional SEM/EDX images of the GDE after 24 h electrolysis, yellow = Bi, pink = K; (b) Raman spectra of the GDE before and after the 24 h electrolysis; and (c) SEM images of the catalyst structure before and after 24 h electrolysis, with (BiO)_2_CO_3_ as the catalyst.

Cross-sectional images show that these K^+^ salts are primarily deposited on the catalyst, with no significant salt accumulation within the internal structure of the GDE, aside from slight penetration of K^+^, likely due to mild perspiration observed during the experiment. Figure 8b confirms that the CL composition remains stable after the 24 h electrolysis, as the catalyst oxidation state remains unchanged, with (BiO)_2_CO_3_ as the main component. However, the Raman peaks appear less intense, probably due to partial coverage of the active site with salt deposits or due to the nanostructure reconstruction, which may imply a crystallinity loss. The *Sustainion* and PTFE peaks also remain unchanged after the experiment. The catalyst structure of the used GDEs after the 24 h experiment is further investigated via SEM imaging, as shown in Figure 8c. As observed, there is a reconstruction of the catalyst, which is also determined after the 8 h electrolysis (Figure 6a). In the images taken after 8 h of experimentation, for the (BiO)_2_CO_3_–*Sustainion–PTFE* 90-(7.5–2.5) composition, it can be observed that the reconstruction and formation of the nanoflowers are incomplete, and their size is smaller than for the case of *Nafion* 70–30 and *Sustainion* 90–10. However, after 24 h under reduction conditions, CO_2_ exposure, and a current density of −200 mA cm^–2^, the nanoflowers appear to be fully formed, resembling those in the other two cases after 8 h. This suggests that the catalyst rearrangement may occur at different rates depending on the CL composition.

Overall, it is demonstrated that the optimization of the CL composition, with a (BiO)_2_CO_3_–*Sustainion–PTFE* ratio of 90-(7.5–2.5), enables the GDE to operate for 24 h while maintaining high FE toward formate and keeping low FE toward H_2_.

## Conclusions

In summary, the role of ionomers in the stability of the CL of GDEs for ERCO_2_ to formate is investigated. First, the effect of changing the catalyst-to-ionomer ratio is evaluated for a cation-conductive ionomer, such as *Nafion*. Therein, low *Nafion* loads, as in the cases of catalyst–*Nafion* 90–10 and 70–30, result in FE toward formate close to 85%, while maintaining FE toward H_2_ below 5%. However, as the ionomer loading increases, a poorer lateral distribution of the catalyst and the blockage of CO_2_ access pathways to active sites leads to a significant reduction in formate FE, down to 11% for the catalyst–*Nafion* ratio of 30–70, favoring the HER, with FE exceeding 60%.

A similar evaluation is conducted for the use of an anion-conductive ionomer. In this case, the nature of the ionomer suppresses the HER for all catalyst–*Sustainion* ratios, achieving the highest FE toward formate for the 90–10 ratio, exceeding 90%. A trend similar to that observed with *Nafion* is found when increasing the ionomer loading, as poorer catalyst distribution and pore clogging reoccur, limiting ERCO_2_ to formate.

The addition of PTFE alongside *Sustainion* is also studied to tune the hydrophobicity of the GDE and improve its stability. In this regard, different *Sustainion–PTFE* proportions are established while maintaining the catalyst–binder ratio at 90–10. The GDE that yields the best results among the investigated ratios is (BiO)_2_CO_3_–*Sustainion–PTFE* 90-(7.5–2.5), achieving FE toward formate of 90% and 96% at current densities of −200 and −300 mA cm^–2^, respectively.

Subsequently, the GDEs that performed best in the screening of the catalyst-to-ionomer ratio are evaluated in 8 h of stability experiments, applying a constant −200 mA cm^–2^ current density. These experiments reveal that the composition of the CL has a significant effect on stability. In the case of the *Nafion* 70–30 and *Sustainion* 90–10 GDEs, a sudden increase in FE toward H_2_ is observed, which is continuously monitored. This indicates that after a certain period, the GDE becomes flooded, limiting CO_2_ access to the catalyst and favoring the HER. However, for the (BiO)_2_CO_3_–*Sustainion–PTFE* 90-(7.5–2.5) GDE, FE toward H_2_ remains below 2% throughout the entire experiment, while an FE toward formate of 91% is achieved.

Given the promising results in the 8 h tests, this GDE, which combines *Sustainion* and PTFE as binders, is tested in a longer 24 h stability experiment. The results are promising, as FE toward H_2_ remains below 10% without sudden increases, indicating that this CL composition prevents GDE flooding. Additionally, high formate production is maintained throughout the period, with FEs reaching nearly 85%. The results of this study demonstrate the significant impact of CL composition on GDE stability. Through systematic screening and optimization using *Sustainion* as the ionomer, adding PTFE to tune hydrophobicity, and maintaining a catalyst–binder ratio of 90–10, the stability of the GDE is extended from hours to a time scale of days. This improvement brings this technology closer to potential scaling by enhancing the long-term stability of GDEs, as this GDE lifetime allows for the transition from laboratory-scale testing to the development of demonstrators or pilot plants to test this ERCO_2_ technology under relevant industrial conditions.

## Methods

### GDE Fabrication

The synthesis of (BiO)_2_CO_3_ nanosheets is carried out by suspending 234 mg of Bi_2_O_3_ (99.9%, Merck KGaA) in 10 mL of deionized H_2_O and dissolving it by stirring after the addition of 3 mL concentrated HNO_3_ (65%, VWR). Precipitation is carried out using 2.5 g of Na_2_CO_3_ (≥97%, VWR) dissolved in 10 mL of deionized H_2_O. This solution was then added to the Bi^3+^ containing one until pH 7 was reached. Afterward, suspension aging took place at 85 °C for 3 h to enforce the crystallization of (BiO)_2_CO_3_. Finally, the white product was filtered off, washed five times with 20 mL of deionized H_2_O, and dried at 70 °C for 12 h.

The different GDEs tested in this study are fabricated using vacuum spray deposition. In this process, the catalytic ink is sprayed onto a commercial GDL (Sigracet 36 BB) by using a manual airbrush. The GDL is placed over a vacuum filtration membrane to ensure proper deposition of the catalytic ink.

The ink is composed of isopropanol (97 wt %) as the solvent, with the catalyst and ionomer suspended in different mass ratios. Two different bibased catalytic materials are employed: synthesized (BiO)_2_CO_3_ nanosheets and commercial Bi_2_O_3_ nanoparticles (Sigma-Aldrich, 90–210 nm).

Two different ionomers are used to bind the catalyst particles to the GDL and facilitate ion conduction: (i) a proton-conductive ionomer, *Nafion* D-521 (Sigma-Aldrich), and (ii) an anion-conductive ionomer, *Sustainion* XC-2 (Dioxide Materials). The catalyst–ionomer ratio is varied systematically, as summarized in Table 1.

**Table 1:** Summary of Ionomer Types and Catalyst/Ionomer Ratios Evaluated

ionomer	catalyst	catalyst/ionomer ratio
*Nafion*	(BiO)_2_CO_3_	90–10
		70–30
		50–50
		30–70
*Sustainion*	(BiO)_2_CO_3_	90–10
		70–30
		50–50
		30–70

In addition to ionomer selection, PTFE (Powder, Sigma-Aldrich) is introduced as an additive to the catalytic ink to adjust the hydrophobicity of the CL. This modification is applied specifically in combination with *Sustainion*, with the total catalyst/ionomer ratio maintained constant, while *Sustainion–PTFE* is systematically varied as follows: 75–25, 50–50, 25–75, and 0–100. The fabricated GDEs have a geometrical active area of 1 cm^2^, with a catalyst loading of 0.75 mg cm^–2^.

Alternative carbon supports are also investigated, including Teflon-coated carbon paper (AvCarb MGL 190 – 50 wt % PTFE-treated) and carbon cloth (CT Carbon cloth W0S1011). For each case, an MPL layer is deposited onto the substrate to enhance the electrode’s structural and transport properties. The MPL is comprised of Vulcan XC-72R (Cabot) and PTFE in a 60–40% wt ratio, with a loading of 2 mg cm^–2^.

### Experimental Setup

The experiments are conducted using a filter-press reactor (ElectroCell) with a 1 cm^2^ active area (Figure 9a). Pure CO_2_ is supplied to the cathode side at a flow rate of 25 mL min^–1^ in a flow-by, single-pass configuration (Figure 9b). The catholyte compartment is separated by the gas diffusion electrode (GDE), with a 0.5 M KHCO_3_ solution recirculated at 7.5 mL min^–1^ throughout the experiment. A cation exchange membrane (PFSA D-50-U DuPont) is used to separate the cathode and anode compartments. The anolyte, consisting of a 1 M KHCO_3_ solution, is also recirculated at 7.5 mL min^–1^. A titanium foil serves as the counter electrode, while a reference electrode (Ag/AgCl 3.5 M) is positioned in the catholyte compartment to enable continuous monitoring of the cathode potential.

**Figure 9:**
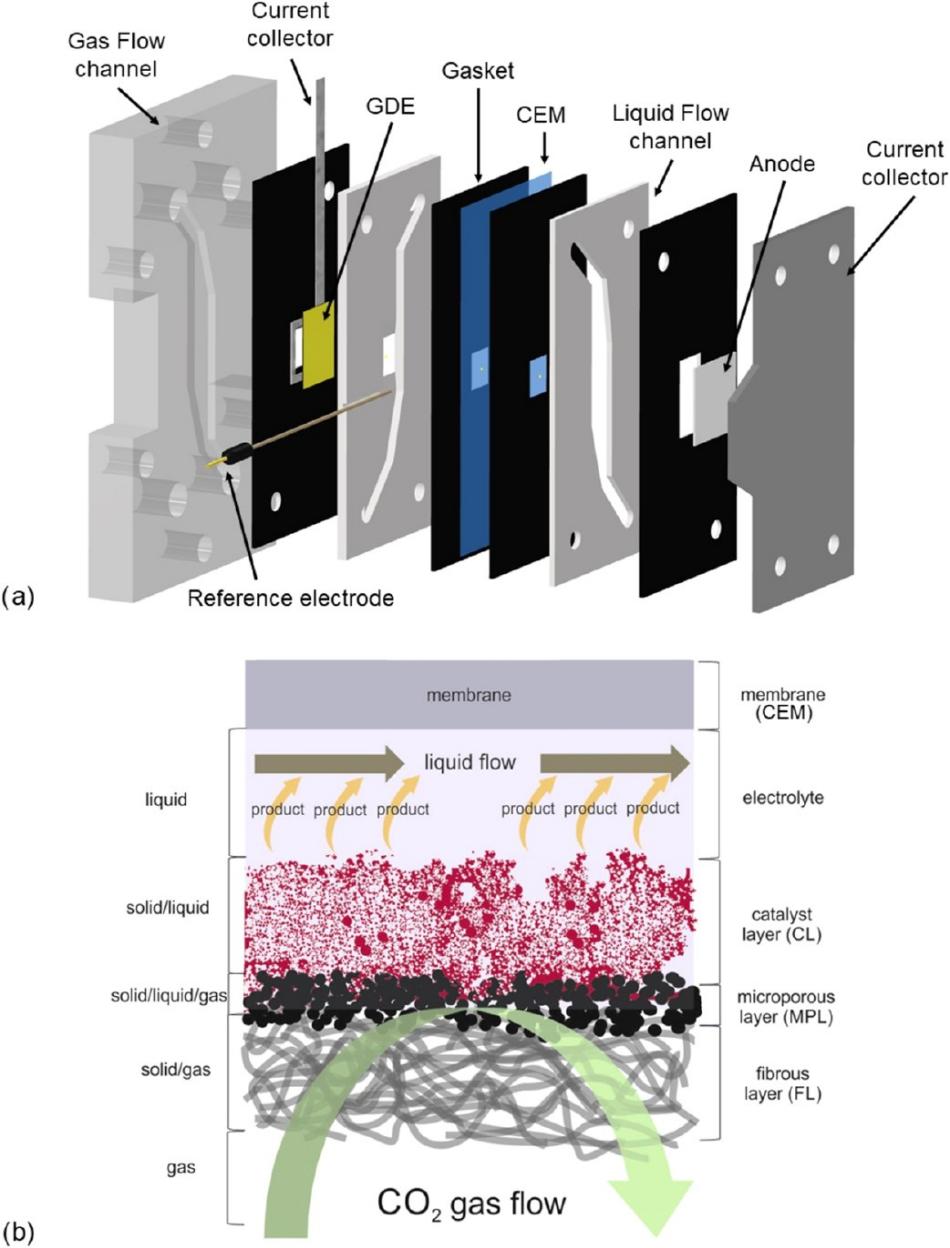
(a) Schematic representation of the filter-press reactor. (b) Diagram illustrating the operation of the GDE in a flow-by configuration.

The experiments are performed in a galvanostatic mode, applying a current density of −200 mA cm^–2^ using a potentiostat (ECi-210, Nordic Electrochemistry). This current density was selected based on technoeconomic studies that identify −200 mA cm^–2^ as an optimal operating point under industrially relevant conditions.^5^ At this value, a favorable balance is achieved between the high Faradaic efficiency for formate and low energy consumption, addressing one of the key challenges for the scalability of CO_2_ electroreduction technologies. To assess additional operational parameters, the CO_2_ inlet pressure (before the reactor) and catholyte side pressure are continuously monitored using pressure sensors (OMEGA PXM309). These pressures are maintained within the following ranges: CO_2_ inlet pressure: 90–110 mbar and catholyte pressure: 0–25 mbar. A moderate pressure difference between the CO_2_ gas inlet and the liquid catholyte phase prevents rapid electrolyte flooding of the GDE, which is observed at the same pressure. It also prevents the formation of CO_2_ bubbles entering the electrolyte phase, which is observed when the gas inlet pressure is too high. In addition, a fixed tightening torque of 4 N m is used in all experiments to ensure uniform mechanical conditions and to allow a standardized, reproducible comparison of different GDE compositions.

To detect potential perspiration or flooding of the catholyte through the GDE into the gas outlet, a conductivity trap is placed at the CO_2_ outlet. Additionally, the pH (781 pH/Ion Meter, Metrohm) and conductivity (CDM210, MeterLab) of the catholyte are continuously recorded. Gaseous products are analyzed every 10 min using gas chromatography (GC, SRI 8610C), while liquid products are quantified postexperiment using ion chromatography (Metrohm 940 Professional IC).

The duration of each experiment varies depending on the evaluation strategy: (i) preliminary screening: 90 min tests are conducted to evaluate different CL compositions, (ii) intermediate stability assessment: the best-performing compositions are tested for 8 h runs, and (iii) long-term stability testing: the most stable composition is evaluated in a 24 h continuous operation test.

The electrode performance is assessed by analyzing the Faradaic efficiency (FE), which indicates the selectivity of the applied external current toward the formation of a specific product, formate rate, and single-pass conversion efficiency (SPCE), which refers to the percentage of CO_2_ converted in a single pass through the electrochemical cell. The corresponding equations are provided in the Supporting Information.

### GDE Characterization

The fabricated GDEs are systematically characterized before and after electrolysis to assess their structural integrity, composition, and surface properties. Structural and compositional analyses are performed using scanning electron microscopy (SEM) for top-down and cross-sectional imaging, coupled with energy-dispersive X-ray (EDX) analysis. These measurements are carried out with a Zeiss DSM 982 SEM equipped with a Noran SIX NSS200 EDX spectrometer. Surface composition is assessed by using Raman spectroscopy with a LabRAM HR800 confocal microscope (Horiba Jobin Yvon). Spectral data are acquired using Lab Space 3.0 software and seamlessly integrated with the Raman spectrometer and confocal microscope for precise analysis.

The hydrophobicity of the as-prepared GDEs is evaluated through contact angle measurements conducted using a DSA25 Krüss Advance Drop Shape Analyzer (Krüss GmbH, Hamburg, Germany). The electrodes are placed on a flat sample stage, and water droplets (1.4 μL of Milli-Q water) are deposited at room temperature.

Furthermore, the physicochemical characterization of the (BiO)_2_CO_3_ catalyst is carried out by XRD and STEM. X-ray diffractograms are measured using a Panalytical X’Pert Pro X-ray diffractometer (Malvern Panalytical GmbH, Kassel, Germany) with the Bragg–Brentano geometry and Cu Kα radiation with a Ni filter. The diffractograms are recorded in the range of 5°–80° over a period of 120 min. The reflections are evaluated using the QualX software (version 2.24)^53,54^ and compared to references from the Crystallography Open Database (COD). To obtain the relative crystalline composition and particle sizes of the samples analyzed by XRD, Rietveld refinement is performed using the software Profex 5.4.1.^55^

Finally, EIS measurements were performed using an AutoLab PGSTAT 302 N instrument (Metrohm Hispania) in a filter-press cell setup. The tests were conducted at a constant potential of −0.8 V vs Ag/AgCl, within a frequency range of 10 kHz to 0.1 Hz, to characterize the surface electrochemical behavior of the GDEs.

## Supplementary Material



## Notes

Figures of merit description, XRD and STEM of the catalyst, EIS of different GDE compositions, additional long-term stability results, and SEM/water contact angle characterization (PDF)
