# Supplementary material for: GDE Stability in CO2 Electroreduction to Formate: The Role of Ionomer Type and Loading
Source: ACS Catal. 2025 May 9;15(11):8753–67. doi: 10.1021/acscatal.5c02052 (PMC12239591; doi:10.1021/acscatal.5c02052)
Supplement: Supplementary file 1 [file cs5c02052_si_001.pdf]

Supporting Information

GDE stability in CO_2 electroreduction to formate: The role of ionomer type and loading

Jose Antonio Abarca[a,*], Lucas Warmuth[b], Alain Rieder[c,d], Abhijit Dutta[c,d], Soma Vesztergom[c,d,e], Peter Broekmann[c,d*], Angel Irabien[a], Guillermo Díaz-Sainz[a]

a Departamento de Ingenierías Química y Biomolecular, Universidad de Cantabria, Avenida de los Castros s/n, 39005 Santander, Spain

b Institute of Catalysis Research and Technology (IKFT), Karlsruhe Institute of Technology (KIT), Hermann-von-Helmholtz-Platz 1, Eggenstein-Leopoldshafen, 76344, Germany

c Department of Chemistry, Biochemistry and Pharmaceutical Sciences, University of Bern, Freiestrasse 3, Bern, 3012, Switzerland

d NCCR Catalysis, University of Bern, Freiestrasse 3, Bern, 3012, Switzerland.

e MTA–ELTE Momentum Interfacial Electrochemistry Research Group, Eötvös Loránd University, Pázmány Péter sétány 1/A, Budapest, 1117, Hungary

*Corresponding autors: joseantonio.abarca@unican.es, peter.broekmann@unibe.ch

- Faradaic Efficiency (FE):

$$FE_{Liquid} = \frac{z \cdot F \cdot V_{catholyte} \cdot c_i}{Q \cdot M_i} \quad (1)$$

$$FE_{Gas} = \frac{z \cdot F \cdot \upsilon \cdot x_i}{I \cdot V_m} \quad (2)$$

Where z is the number of exchanged electrons, F is the Faraday constant (96485 C mol^{-1}), $V_{catholyte}$ is the total volume of the recirculated catholyte, c_i is the liquid product concentration (ppm), Q is the total charge, M_i is the molar mass of the liquid product, υ is the volumetric gas flowrate, x_i is the molar fraction of the gas product, and V_m is the molar volume of a gas at ambient temperature and pressure.

- Formate rate, *r*, evaluates the rate of formate production per unit area and time:

$$r\left(\frac{mmol}{m^2\, s}\right) = \frac{M}{t \cdot A} \quad \textbf{(3)}$$

Where M and A are as defined above, and t is the duration of the experiment.

- Single-pass conversion efficiency, SPCE, represents the fraction of CO_2 converted into formate during a single pass through the electrochemical reactor.

$$SPCE\ (\%) = \frac{\frac{j \cdot A \cdot t \cdot FE}{z \cdot F} \cdot V_{mol}}{V_{IN} \cdot t} \quad \textbf{(4)}$$

Where j, A, t, z, F, and FE are as defined, V_{mol} is the molar volume of an ideal gas under standard conditions (22.4 L), and V_{IN} is the volumetric flowrate of CO_2 (L min^{-1}).

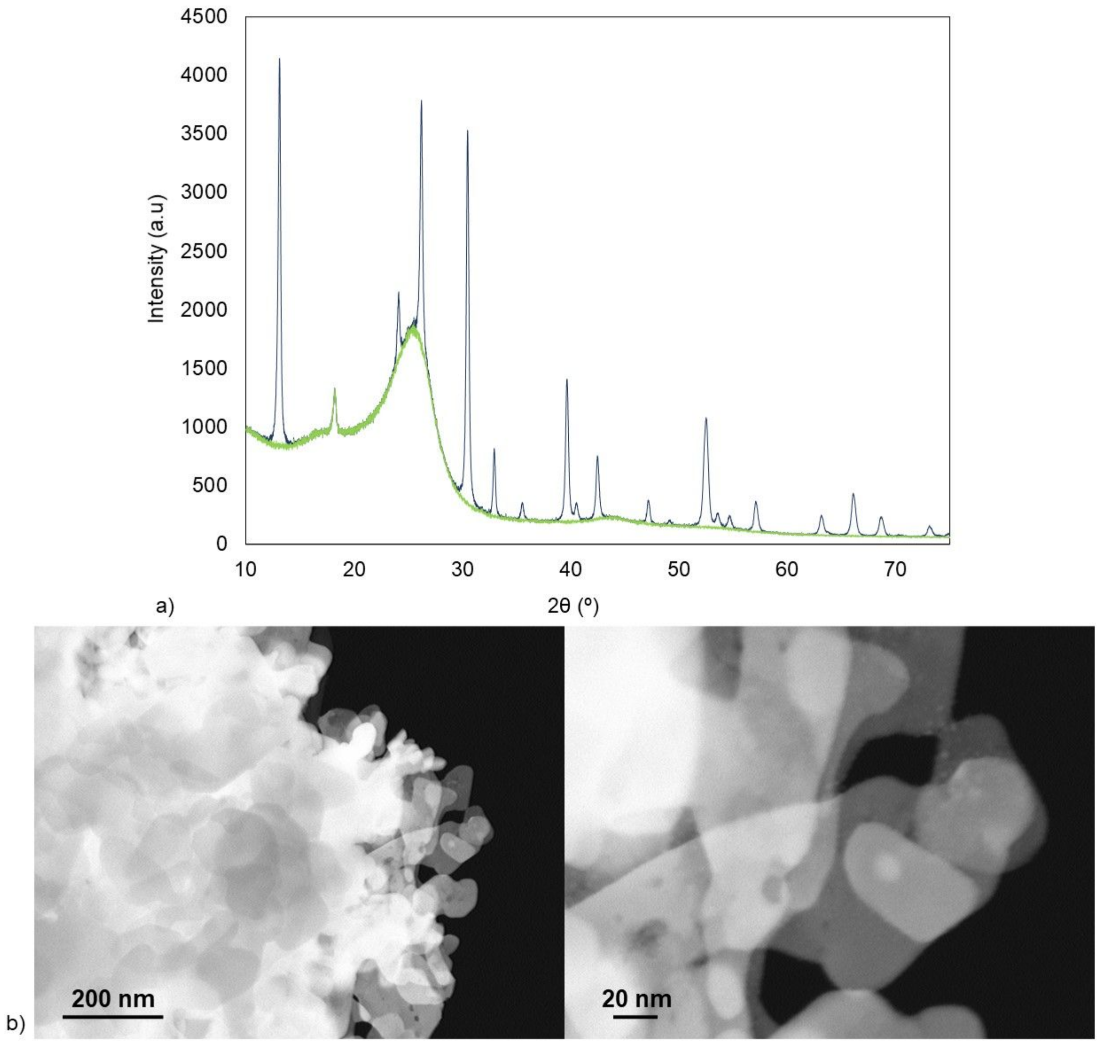

Figure S1. A) XRD analysis diffractogram for the as-prepared $(BiO)_2CO_3$ GDE surface (blue) and the GDE background (green), and b) STEM images of the $(BiO)_2CO_3$ powder at 50kX and 200kX magnification.

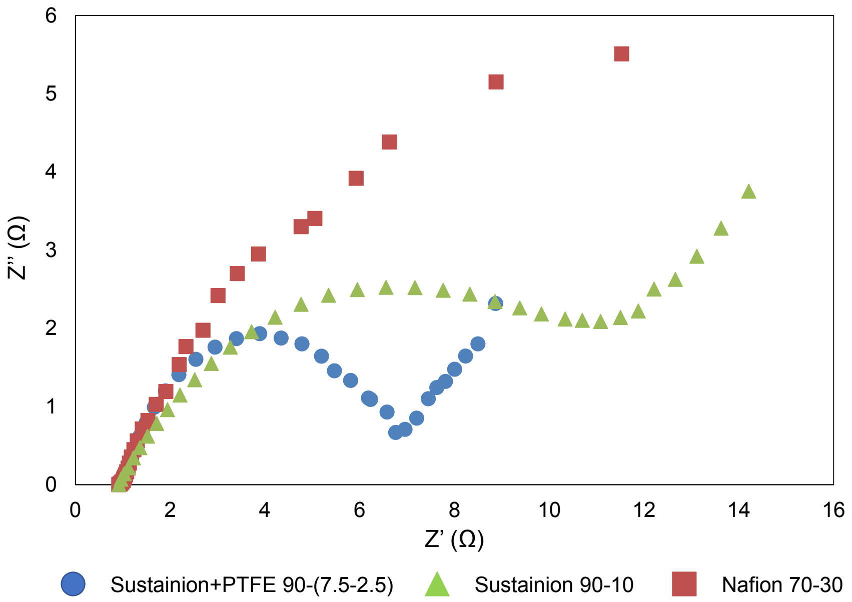

Figure S2. Nyquist plot for EIS results for the different GDE evaluated at -0.8 V (vs. Ag/AgCl) in a frequency range from 10K Hz to 0.1 Hz.

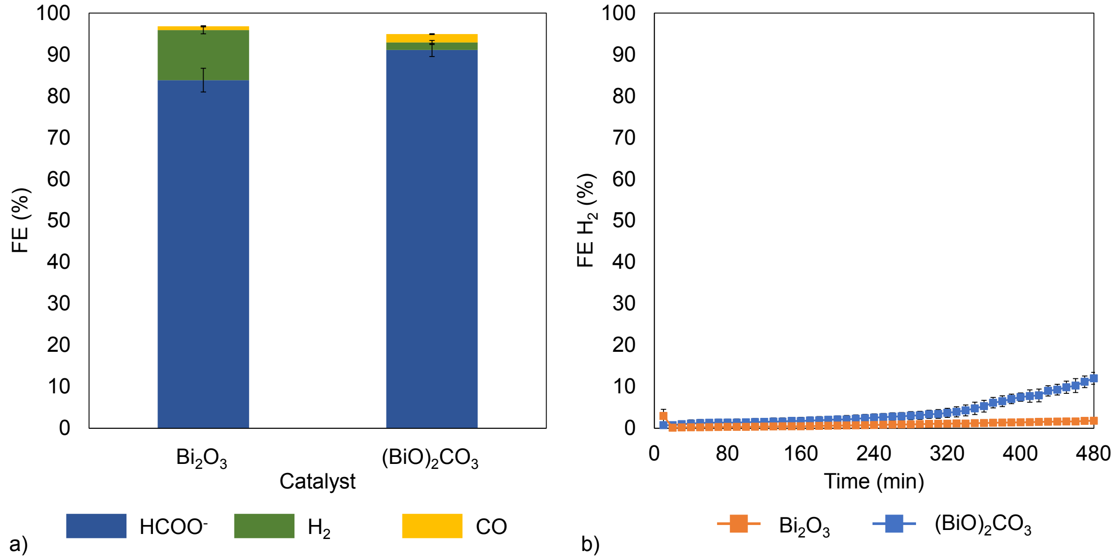

Figure S3. a) Comparison of the FE for different reaction products, and b) continuous monitoring of H_2 FE over 8 hours of electrolysis using Bi_2O_3 and $(BiO)_2CO_3$ catalysts.

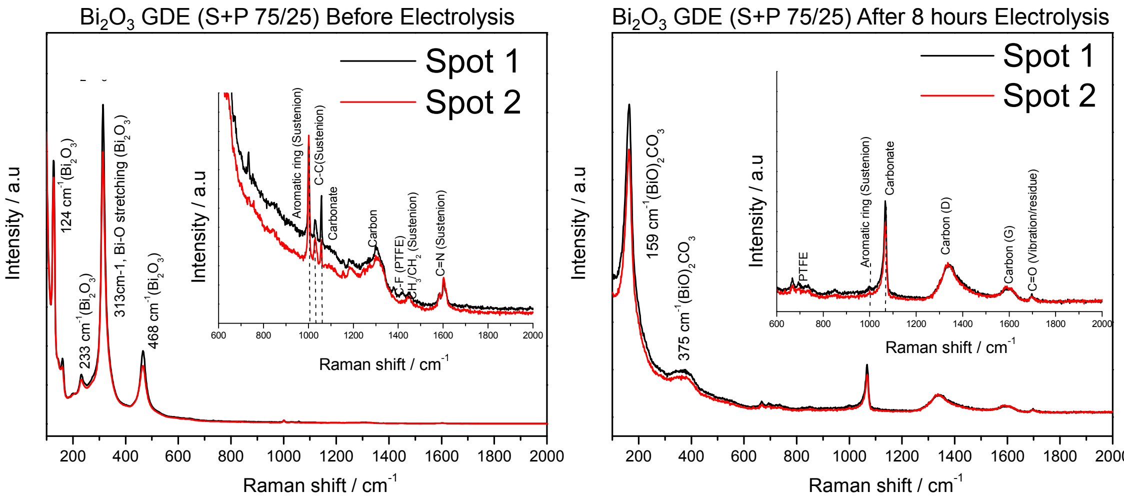

Figure S4. Raman spectra of the Bi_2O_3-based GDE before and after 8 hours of electrolysis.

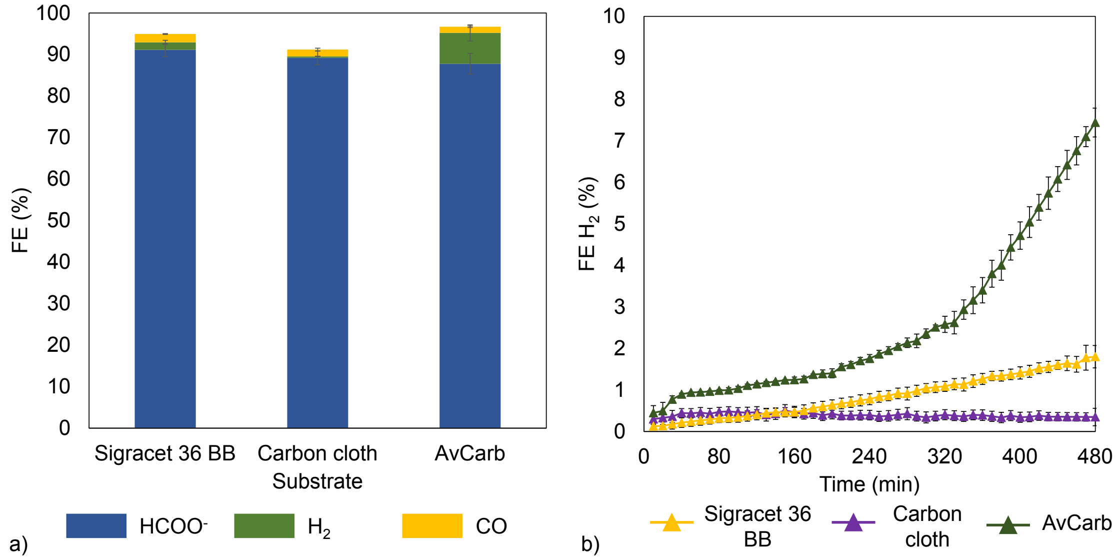

Figure S5. a) FE of different substrates, and b) FE H_2 monitoring during the 8 hours of experimental time.

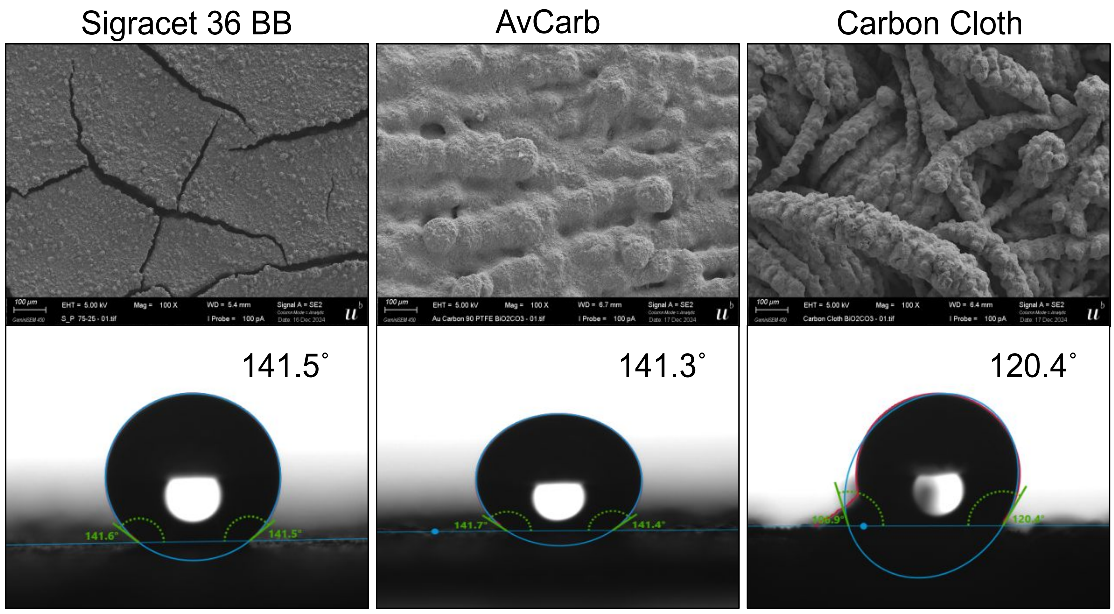

Figure S6. Top-down SEM images and water contact determination for the GDEs with different substrates.

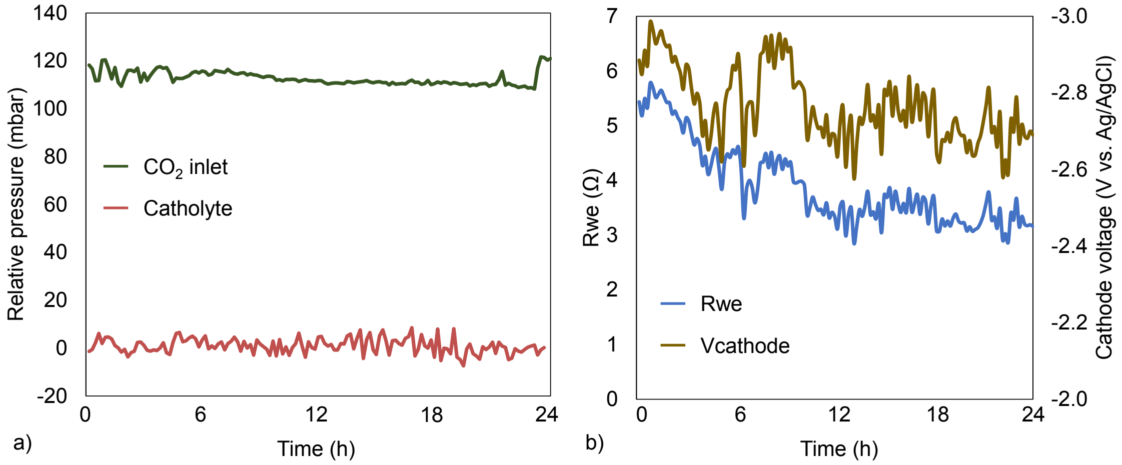

Figure S7. Evolution during the 24-hour experiment of a) Pressure difference between the CO_2 inlet and the catholyte, and b) Working electrode resistance and cathode voltage.

Table S1. Summary of the different figures of merit evaluated for the various GDE compositions:

GDE	Time (h)	CO_2 flowrate (mL min^{-1})	Current density (mA cm^{-2})	Formate concentration (ppm)	Formate produced (mmol)	FE (%)	Rate (mmol s^{-1} m^{-2})	SPCE (%)
Nafion 90-10	1.5	200	200	2367	4.76	84.8	8.82	5.91
Nafion 70-30	1.5	200	200	2386	4.72	84.3	8.74	5.87
Nafion 50-50	1.5	200	200	1820	3.74	66.8	6.93	4.65
Nafion 30-70	1.5	200	200	304	0.62	11.2	1.16	0.78
Sustainion 90-10	1.5	200	200	2523	5.13	91.6	9.50	6.38
Sustainion 70-30	1.5	200	200	2671	5.02	89.6	9.29	6.24
Sustainion 50-50	1.5	200	200	1920	3.82	68.2	7.07	4.75
Sustainion 30-70	1.5	200	200	1861	3.52	62.6	6.51	4.36
S+P 90-7.5-2.5	1.5	200	200	3065	5.11	88.9	9.46	6.19
S+P 90-5-5	1.5	200	200	2324	5.04	89.8	9.32	6.25
S+P 90-2.5-7.5	1.5	200	200	2416	5.07	90.6	9.40	6.31
S+P 90-0-10	1.5	200	200	2504	5.01	89.5	9.27	6.23
Sustainion 90-10	1.5	200	300	4248	8.02	95.6	14.86	9.98
S+P 90-7.5-2.5	1.5	200	300	4006	8.10	96.5	15.00	10.08
S+P 90-5-5	1.5	200	300	3935	7.08	86.4	13.12	9.02
S+P 90-2.5-7.5	1.5	200	300	3651	7.30	87.0	13.52	9.09
S+P 90-0-10	1.5	200	300	4019	6.88	81.9	12.74	8.56
Nafion 70-30	8	200	200	8130	13.55	45.4	4.70	3.16
Sustainion 90-10	8	200	200	8730	15.91	53.1	5.52	3.70
S+P 90-7.5-2.5	8	200	200	12618	27.20	91.1	9.44	6.35
S+P 90-7.5-2.5	24	200	200	25305	77.04	83.4	8.92	5.81
Diaz-Sainz et al. 2019	1.5	200	200	3950	-	80.4	8.33	5.60
Abarca et al. 2023	1.5	200	200	4750	-	96.7	10.01	6.73
